# Glycomacropeptide: long-term use and impact on blood phenylalanine, growth and nutritional status in children with PKU

**DOI:** 10.1186/s13023-019-1011-y

**Published:** 2019-02-15

**Authors:** A. Daly, S. Evans, S. Chahal, S. Santra, A. Pinto, R. Jackson, C. Gingell, J. Rocha, F. J. Van Spronsen, A. MacDonald

**Affiliations:** 1 Dietetic Department, Birmingham Childrens Hospital, Steelhouse Lane, Birmingham, B4 6 NH UK; 2 University of Liverpool, Brownlow Street, Liverpool, L69 3GL UK; 3 Nottingham Queen’s Medical Centre, University Hospital, Derby Road, Nottingham, NG7 2UH UK; 4 Centro de Genética Médica JM, CHP EPE, Porto, Portugal; 5 Centro de Referência na área das Doenças Hereditárias do Metabolismo, Centro Hospitalar do Porto – CHP EPE, Porto, Portugal; 6 Faculdade de Ciências da Saúde, UFP, Porto, Portugal; 7 Center for Health Technology and Services Research (CINTESIS), Porto, Portugal; 8 Beatrix Children’s Hospital, University Medical Centre of Groningen, University of Groningen, Groningen, The Netherlands

**Keywords:** Glycomacropeptide, Phenylalanine, Large neutral amino acids, Protein substitute, Phenylketonuria

## Abstract

**Abstract:**

In phenylketonuria, casein glycomacropeptide (CGMP) requires modification with the addition of some essential and semi essential amino acids to ensure suitability as a protein substitute. The optimal amount and ratio of additional amino acids is undefined.

**Aim:**

A longitudinal, parallel, controlled study over 12 months evaluating a CGMP (CGMP-AA2) formulation compared with phenylalanine-free L-amino acid supplements (L-AA) on blood Phe, Tyr, Phe:Tyr ratio, biochemical nutritional status and growth in children with PKU. The CGMP-AA2 contained 36 mg Phe per 20 g protein equivalent.

**Methods:**

Children with PKU, with a median age of 9.2 y (5-16y) were divided into 2 groups: 29 were given CGMP-AA2, 19 remained on Phe-free L-AA. The CGMP-AA2 formula gradually replaced L-AA, providing blood Phe concentrations were maintained within target range. Median blood Phe, Tyr, Phe:Tyr ratio and anthropometry, were compared within and between the two groups at baseline, 26 and 52 weeks. Nutritional biochemistry was studied at baseline and 26 weeks only.

**Results:**

At the end of 52 weeks only 48% of subjects were able to completely use CGMP-AA2 as their single source of protein substitute. At 52 weeks CGMP-AA2 provided a median of 75% (30–100) of the total protein substitute with the remainder being given as L-AA. Within the CGMP-AA2 group, blood Phe increased significantly between baseline and 52 weeks: [baseline to 26 weeks; baseline Phe 270 μmol/L (170–430); 26 weeks, Phe 300 μmol/L (125–485) *p* = 0.06; baseline to 52 weeks: baseline, Phe 270 μmol/L (170–430), 52 weeks Phe 300 μmol/L (200–490), *p* < 0.001)]. However, there were no differences between the CGMP-AA2 and L-AA group for Phe, Tyr, Phe:Tyr ratio or anthropometry at any of the three measured time points. Within the CGMP-AA2 group only weight (*p* = 0.0001) and BMI z scores (p = 0.0001) increased significantly between baseline to 52 weeks. Whole blood and plasma selenium were significantly higher (whole blood selenium [*p* = 0.0002]; plasma selenium [*p* = 0.0007]) at 26 weeks in the CGMP-AA2 group compared L-AA. No differences were observed within the L-AA group for any of the nutritional markers.

**Conclusions:**

CGMP-AA increases blood Phe concentrations and so it can only be used partly to contribute to protein substitute in some children with PKU. CGMP-AA should be carefully introduced in children with PKU and close monitoring of blood Phe control is essential.

**Electronic supplementary material:**

The online version of this article (10.1186/s13023-019-1011-y) contains supplementary material, which is available to authorized users.

## Introduction

The composition, balance and ratio of amino acids in protein substitutes for phenylketonuria (PKU) requires further consideration and study. Protein substitutes not only provide 50–80% of the total nitrogen supply for growth; they also have a number of physiological properties potentially influencing phenylalanine (Phe) concentrations and anabolism [1, 2]. The delivery of amino acids (AA) into the systemic circulation alters their plasma ratio affecting the uptake of AA at both brain [3–5] and gut membranes [6]. Protein substitutes also contain higher amounts of leucine compared with cow’s milk protein (mean amount per 10 g protein equivalent: L-AA, 127 mg ± 23 mg; cow’s milk, 100 ± 10 mg). Leucine stimulates muscle protein synthesis and anabolism by activation of a complex pathway involving mTOR (mammalian target of rapamycin), which stimulates insulin secretion [7]. However, there is evidence that L-AA are absorbed more rapidly than intact protein sources that requires digestion leading to less AA retention [8, 9] whereas low Phe glycomacropeptide (CGMP) containing a peptide component may be more efficacious [10]. CGMP is a ‘residue’ peptide found in the extracted whey component of cheese. Although it is high in some large neutral amino acids (LNAA) such as threonine and isoleucine, it is low in several essential AA and tyrosine, and so requires supplementation to ensure suitability for use in PKU.

Protein substitutes are essential in the management of PKU, but the majority are based on mono amino acids. Some children struggle accepting the taste, smell and prescribed daily volume, while others complain of abdominal discomfort and bad breath. CGMP a peptide-based substitute with added amino acids offers an alternative choice to the traditional mono amino acid substitutes. Identifying suitable alternative protein substitutes with acceptable palatability and improved biological efficacy is important in the treatment of PKU.

Commercial CGMP protein substitutes (supplemented with L-amino acids) (CGMP-AA) contain at least 36 mg of Phe per 20 g protein equivalent (Vitaflo International Ltd.). We have previously reported the impact of CGMP-AA1 on blood Phe concentrations in a group of children aged 5–16 years of age using a formula containing 30 mg of Phe per 20 g protein equivalent [11]. Although, the blood Phe concentrations remained within the target reference range, they increased significantly compared with a control group who remained on L-AA. Therefore, we concluded that CGMP-AA1 could only partially replace the L-AA in most children. We also considered that the blood Phe control might be improved by using a CGMP-AA with a higher concentration of some essential and conditionally essential AA comparable to conventional L-AA supplements. Therefore, we made small adjustments to the AA formulation of the CGMP-AA, so they were similar in profile to conventional L-AA supplements.

In this paper, we explain the changes made to the AA profile of a CGMP-AA formula and the impact this had on blood Phe, Tyr, and Phe:Tyr ratio. As CGMP has not been extensively used as a protein substitute in children, we also investigated changes in the biochemical nutritional status and growth in children using modified CGMP-AA compared to conventional protein substitutes. Therefore, in a 12-month longitudinal, prospective study, we describe the impact of using this modified CGMP-AA formulation (CGMP-AA2) compared with a control group of children taking conventional Phe-free L-AA supplements only.

## Methods

### Subjects

Fifty children (28 boys, 22 girls) with PKU were recruited. Their median age at recruitment was 9.2 years (range 5-16y). Forty-seven children were European and 3 were of Pakistani origin. Inclusion criteria included: diagnosed by newborn screening, aged 5 to 16 years, not treated with sapropterin dihydrochloride, known adherence with protein substitute, and 70% of blood Phe concentrations within Phe target range for 6 months prior to study entry. Target blood Phe ranges for children aged 5 to 12 years were < 360 μmol/L and for 12 years and older < 600 μmol/L according to the European guideline recommendations [12]. Based on untreated blood Phe levels at newborn screening and dietary Phe tolerance, two children in the CGMP-AA2, and one child in the L-AA group had mild PKU with the majority having classical PKU.

The study was registered by the Health Research Authority and was given a favourable ethical opinion by the South Birmingham Research Ethical Committee. Written informed consent was given for all subjects by at least one caregiver with parental responsibility and written assent was obtained from the subjects if appropriate for their age and level of understanding.

### CGMP-AA formulations (Table 1)

CGMP-AA2 is a berry or vanilla flavoured powdered protein substitute containing 20 g of protein equivalent, and 36 mg of Phe per 35 g sachet. Each sachet was mixed with 120 ml of water or phenylalanine-free milk replacement.

**Table 1:** The nutrient composition of CGMP-AA1 and CGMP-AA2 compared with conventional Phe-free L-AA

Nutrients	Units	CGMP-AA1		CGMP-AA2		Phe-free L-AA*	
		Pilot study Week 0–26		Week 0–52		Week 0–52	
		Per 100 g	Per 20 g PE sachet	Per 100 g	Per 20 g PE sachet	Per 100 ml	Per 20 g PE pouch
Calories	Kcal	342	120	342	120	71	124
Protein equivalent	g	57.1	20	57.1	20	11.5	20
Total Carbohydrate	g	18.6	6.5	18.6	6.5	5.4	9.4
Sugars	g	6.3	2.2	6.3	2.2	4.5	7.8
Total Fat	g	4.3	1.5	4.3	1.5	0.4	0.7
DHA	mg	240	84	240	84	77	134
AA	mg	–	–	–	–	–	–
Fibre	g	0	0	0.3	0.1	–	–
Salt	g	1.5	0.53	1.5	0.53	0.25	0.43
Vitamins and minerals							
Vitamin A	μg RE	809	283	809	283	160	278
Vitamin D	μg	12.9	4.5	12.9	4.5	5.8	10
Vitamin E	mg αTE	18.6	6.5	18.6	6.5	3	5.2
Vitamin C	mg	109	38	109	38	21	36
Vitamin K	μg	100	35	100	35	19	34
Thiamine	mg	1.9	0.68	1.9	0.68	0.4	0.7
Riboflavin	mg	2.2	0.78	2.2	0.78	0.44	0.77
Niacin	mg	24	8.4	24	8.4	4.8	8.4
Vitamin B6	mg	2.9	1.0	2.9	1.0	0.5	0.9
Folic Acid	μg	389	136	389	136	77	134
Vitamin B12	μg	4.6	1.6	4.6	1.6	0.9	1.6
Biotin	μg	183	63.9	183	63.9	36	63
Pantothenic acid	mg	7.7	2.7	7.7	2.7	1.5	2.6
Choline	mg	583	204	583	204	115	201
Sodium	mmol	25.8	9.0	25.8	9.0	4.2	7.3
Potassium	mmol	19.3	6.8	19.3	6.8	4.5	7.9
Chloride	mmol	0.6	0.2	0.6	0.2	3.9	6.8
Calcium	mg	1163	407	1163	407	230	400
Phosphorus	mmol	34.2	12	34.2	12	6.6	11.4
Magnesium	mg	366	128	366	128	72	125
Iron	mg	20.9	7.3	20.9	7.3	4.2	7.3
Copper	μg	2137	748	2137	748	420	730
Zinc	mg	20.9	7.3	20.9	7.3	4.2	7.3
Manganese	mg	3.1	1.1	3.1	1.1	0.6	1
Iodine	μg	245	85.7	245	85.7	49	84
Molybdenum	μg	140	49	140	49	28	48
Selenium	μg	85.4	29.9	85.4	29.9	17	29
Chromium	μg	85.4	29.9	85.4	29.9	17	29
L-amino acids							
L-Alanine	g	2.17	0.76	2.23	0.78	0.53	0.92
L-Arginine	g	2.86	1.00	2.71	0.95	0.86	1.5
L-Aspartic Acid	g	5.83	2.04	3.20	1.12	1.36	2.37
L-Cystine	g	0.03	0.01	0.03	0.01	0.35	0.61
L-Glutamine	g	7.11	2.49	7.34	2.57		
Glycine	g	7.91	2.77	3.43	1.2	1.35	2.35
L-Histidine	g	1.20	0.42	2.00	0.7	0.53	0.92
L-Isoleucine	g	3.91	1.37	3.86	1.35	0.93	1.62
L-Leucine	g	3.71	1.30	8.57	3.00	1.46	2.54
L-Lysine	g	3.06	1.07	2.29	0.80	0.96	1.67
L-Methionine	g	1.54	0.54	0.80	0.28	0.26	0.45
L-Phenylalanine	g	0.09	0.03	0.09	0.03	–	–
L-Proline	g	4.31	1.51	4.34	1.52	0.97	1.69
L-Serine	g	2.80	0.98	2.74	0.96	0.6	1.04
L-Threonine	g	6.20	2.17	6.29	2.2	0.93	1.62
L-Tryptophan	g	0.49	0.17	1.14	0.40	0.29	0.5
L-Tyrosine	g	2.89	1.01	6.40	2.24	1.37	2.38
L-Valine	g	3.23	1.13	3.11	1.09	1.07	1.86

*protein substitute based on 20 g PE of PKU Cooler 20 (Vitaflo International Ltd)

§ L- Phenylalanine, CGMP-AA1 0.086g /100g; 0.030g/20g PE (30mg /sachet) CGMP-AA2 0.100g/100g; 0.036g/20g PE (36mg/sachet)

CGMP-AA2 was modified following the findings of a pilot study in which CGMP-AA1 was used. Additional Tyr, tryptophan, leucine and histidine (but less methionine and lysine) were added to provide a similar AA profile to conventional Phe-free L-AA supplements. For each 1 g of protein equivalent, it contained 112 mg Tyr, 20 mg tryptophan, 150 mg leucine, and 35 mg histidine. Lysine and methionine were reduced compared with CGMP-AA1 but still provided minimum amino acid requirements suggested by the WHO/FAO/UNU 2007 [13]. A further difference in the formulation of CGMP-AA2, was the residual Phe content. Due to the manufacturing process of CGMP, there was a 17% increase in residual Phe and CGMP-AA2 contained 36 mg Phe compared to CGMP-AA1 containing 30 mg Phe for every 20 g protein equivalent. No changes were made to the composition of carbohydrate, fat and micronutrients of the CGMP-AA2 throughout the study. Vitaflo International Ltd. produced the CGMP-AA2 study formulation.

#### Control group

Children in the control group remained on their usual Phe-free L-AA, during the study period, with no changes to the AA formulations throughout the study.

### Selection into control or CGMP-AA2 group

Children chose the product they preferred: CGMP-AA2 or Phe-free L-AA. They remained on this formula and in these groups for the duration of the study.

### Study design (Fig. 1)

After completion of the pilot study, and following the changes made to the CGMP formulation, a total of 50 children were recruited and followed up for 12 months. This paper reports the prospective results of Phe and Tyr blood concentrations, Phe:Tyr ratio, nutritional biochemistry and growth (weight, height and BMI z scores) over 12 months, using modified CGMP-AA2 compared to Phe-free L-AA protein substitutes. At baseline and 26 weeks, morning fasted pre-prandial venous samples were collected for nutritional markers. Anthropometry, height and weight together with a stock check of protein substitute usage, diet history, and food frequency questionnaire were collected monthly. All baseline measurements were collected when the children were on Phe-free L-AA. Baseline data for Phe and Tyr blood concentrations, and Phe:Tyr ratio were calculated as a median value from the previous 12 months before study commencement. Data for anthropometry and nutritional biochemistry were collected at the start of the study.

**Fig. 1:**
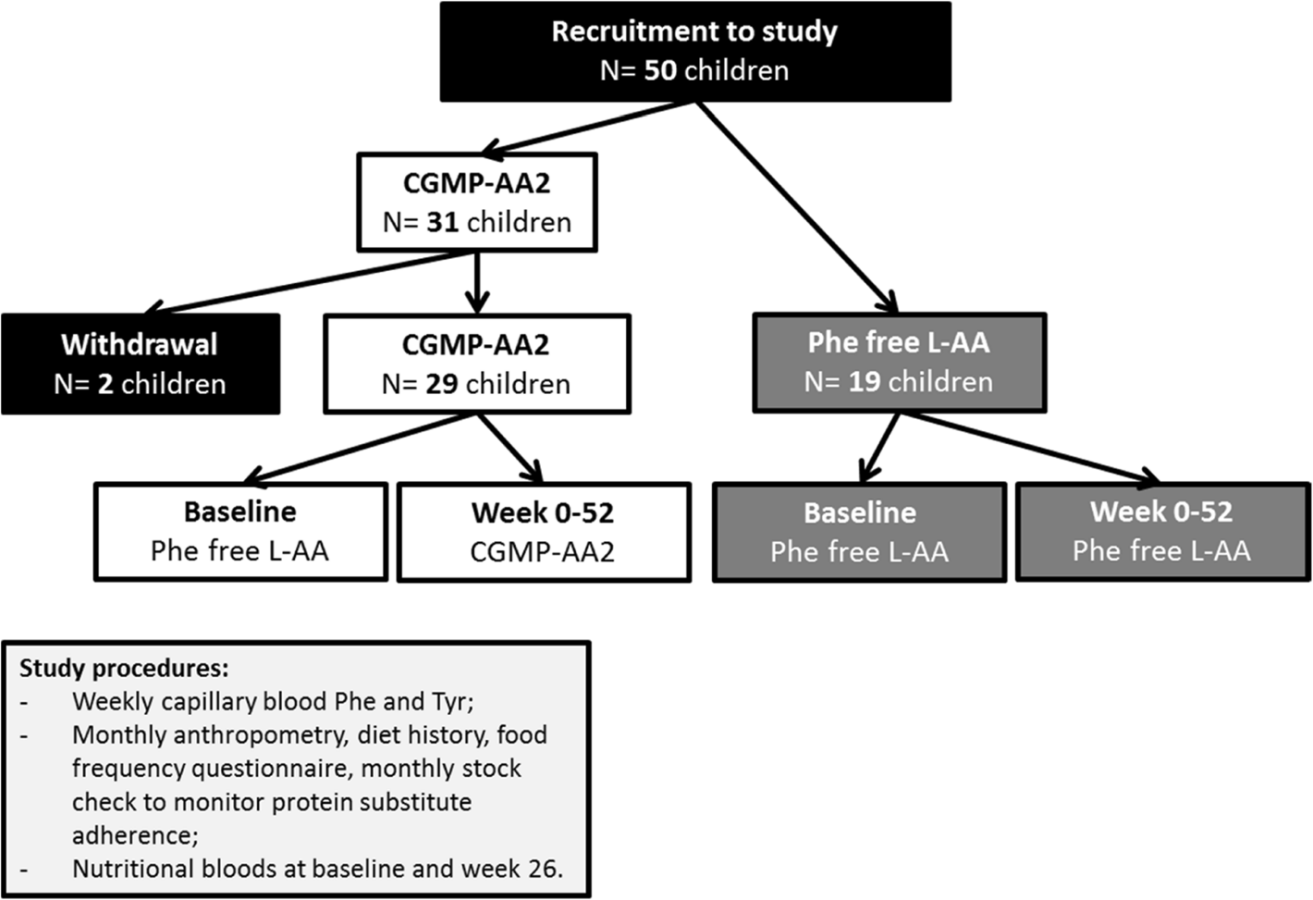
Diagrammatic scheme showing recruitment and introduction of CGMP-AA2 and Phe-free L-AA

### Titration of CGMP-AA2 in the diet

Taking into consideration that CGMP-AA2 contained Phe (36 mg for each 20 g protein equivalent), the dose of CGMP-AA2 was individually prescribed and titrated with the blood Phe concentrations. In the CGMP-AA2 group all the children started with a minimum of 20 g of protein equivalent from CGMP-AA2, with Phe-free L-AA providing the remainder of protein substitute intake. If blood concentrations were stable or decreased, CGMP-AA2 was increased by 20 g per day protein equivalent reducing the Phe-free L-AA by the same amount of protein. If there was any suggestion that blood Phe control deteriorated (criteria blood Phe ≥ target range for 3 consecutive weeks), the dose of CGMP–AA2 was reduced, with a concomitant increase in Phe-free L-AA providing the remaining total protein equivalent requirement. No change or reduction was made to the dietary Phe intake of subjects on the CGMP-AA2.

### Nutritional markers

At baseline and 26 weeks, a fasting morning venous blood sample was collected and analysed for zinc, selenium (plasma and whole blood), calcium, magnesium, phosphate, C-reactive protein (CRP) haemoglobin, MCV (mean cell volume), ferritin, vitamin B12 and 25-hydroxy vitamin D. The laboratory received all samples within 30 min of collection preventing any deterioration in sample quality. All the samples were stored under appropriate conditions and then measured at the same time point, minimising laboratory variations in measurement techniques.

Zinc and selenium (whole blood and plasma) were collected in a lithium heparin ‘trace metal’ tube and analysed using inductively coupled plasma mass spectrometry. Vitamin D was measured as a clotted sample via mass spectrometry. Calcium, magnesium, phosphate, and CRP were all measured in zinc free lithium heparin tubes and measured on a Roche photometric analyser. Ferritin and vitamin B12 were collected in Vacuette Z serum clotted activator tubes and analysed by an Access analyser using electroluminescence. Haemoglobin and MCV were collected into EDTA tubes and measured on a Sysmex XN 2000 analyser.

### Blood Phe/Tyr monitoring

Throughout the study, trained caregivers collected weekly early morning fasted blood spots at home. Blood specimens were sent via first class post to the laboratory at Birmingham Children’s Hospital. Blood samples were collected on filter cards, Perkin Elmer 226 (UK Standard NBS). All the cards had a standard thickness and the blood Phe and Tyr concentrations were calculated on a 3.2 mm punch by MS/MS tandem mass spectrometry.

### Anthropometry, dietary recall and monitoring

Monthly weight and height measurements were collected using portable Seca scales and stadiometer. A mean of three readings were taken and measured to one decimal point. Home visits were completed monthly, collecting a dietary history and food frequency questionnaire, together with adherence monitoring of protein substitute usage by performing a stock check of the amount of protein substitute used. Three trained dietitians measured and assessed the children each month.

### Statistics

Comparisons of Phe data across time were carried out using a linear mixed models approach, which account for both within and between subject levels of variance. Models are constructed which include terms for ‘time’ (Baseline, 26 weeks and 52 weeks), treatment effect (CGMP-AA2 and L-AA) and their interaction. The subject identifier is included as a random effect. Phe is included on the square root scale to ensure normality of model residuals. Results are presented in terms of the mean effect at each time point with associated 95% confidence intervals. Differences between time points and groups are obtained using analysis of deviance tables. All analyses are carried out using programme R (Version 3).

Statistical analysis for nutritional biochemistry and anthropometrics was performed within the groups using non-parametric Wilcoxon matched pairs signed rank test. When comparisons were made between the groups non-parametric Mann Whitney test was performed. Nutritional parameters were measured at baseline and 26 weeks only, anthropometry at baseline, 26 and 52 weeks.

## Results

### Subjects

Of the 50 recruited children, 31 were in the CGMP-AA2 group and 19 in the L-AA control group. Prior to the study start: 6 subjects took powdered protein substitutes [XP Maxamum (Nutricia Ltd.), *n* = 1; PKU Anamix first spoon (Nutricia Ltd.), *n* = 3; PKU gel (Vitaflo International Ltd.), *n* = 2]; and 44 subjects took liquid pouches [PKU Lophlex LQ (Nutricia Ltd.), n = 3; PKU Cooler (Vitaflo International Ltd.), *n* = 41]. In the L-AA group, they either received liquid pouches [PKU Lophlex LQ (Nutricia Ltd.), n = 2; PKU Cooler (Vitaflo International Ltd.), *n* = 14] or powdered preparations [PKU gel (Vitaflo International Ltd.), n = 3]. At the start of the study, the median age was 8.4 years (5–16) in the CGMP-AA2 group, and 11.1 years (5–15) in the L-AA group. Median Phe concentrations at baseline for CGMP-AA2 were 270 μmol/L (170–430) and for L-AA, 315 μmol/L (140–600).

The total median daily dose of protein equivalent before and throughout the study in both groups for protein substitute was 60 g/day (range, 40-60 g). The median number of prescribed protein exchanges was 5 g protein/day (range 3-30 g) or 250 mg Phe (range, 150-1500 mg).

### Subject withdrawal

One boy and one girl (aged 12 years) in the CGMP-AA2 group were withdrawn from the study, both failed to comply with the study protocol, one failing to return blood Phe samples and both had poor adherence with a low Phe diet. A total of 48 children completed the study; 29 in the CGMP-AA2 and 19 in the Phe-free L-AA group.

**Fig. 2:**
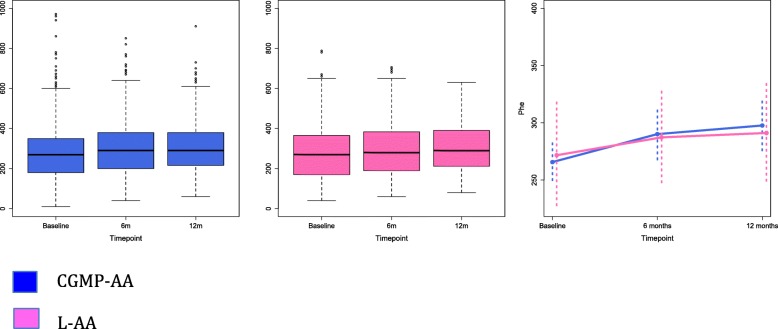
Measured Phe levels in children < 12 years of age in the CGMP-AA and L-AA groups and mean 95% confidence intervals

**Comparison of blood Phe, Tyr, Phe:Tyr ratio between CGMP-AA2 and Phe-free L-AA groups and within groups at: baseline, week 26 and 52 (**Tables 2, 3, 4**)** [Additional file 1. This file shows four additional diagrams, Figures S1A, B, C and S2. Supplementary figures show a mixed linear model for phe, tyr and phe: tyr ratio at baseline, 26 and 52 weeks for all subjects, and Fig. 2 shows phe for subjects< 12 y].

**Table 2:** Changes in the median (range) blood Phe concentrations (μmol/L) within and between the CGMP-AA2 and Phe-free L-AA group at baseline, 26 and 52 weeks

Group	Number of subjects	Baseline μmol/L (range)	Wk 26 μmol/L (range)	Wk 52 μmol/L (range)	*P* value
CGMP-AA2	29	270 ^§*^ (170–430)	300^§^ (125–485)	300^*^ (200–490)	^§^p = 0.06 ^*^p = < 0.0011
Phe-free L-AA	19	315 ^§§**^ (140–600)	325^§§^ (180–580)	340^**^ (190–600)	^§§^*P* = 0.687 ^**^p = 0.236

Wilcoxon test § GMP baseline to 26 wk., * GMP baseline to 52 wk

Wilcoxon test §§ L-AA baseline to 26 wk., ** L-AA baseline to 52 wk

ABREVIATIONS: *CGMP-AA* low phenylalanine glycomacropeptide protein substitute supplemented with L-amino acids, *Phe-free L-AA* Phenylalanine free L-amino acid supplement

**Table 3:** Changes in the median (range) blood Tyr concentrations (μmol/L) within and between the CGMP-AA2 and Phe-free L-AA group at baseline, 26 and 52 weeks

Group	Number of subjects	Baseline μmol/L (range)	Wk 26 μmol/L (range)	Wk 52 μmol/L (range)	P value
CGMP-AA2	29	50^§*^ (30–180)	50^§^ (30–210)	50^*^ (30–170)	^§^*p* = 0.916 ^*^p = 0.239
Phe-free L-AA	19	40^§§**^ (40–100)	40^§§^ (30–105)	40^**^ (30–80)	^§§^*p* = 0.672 ^**^p = 0.111

Wilcoxon test § GMP baseline to 26 wk., * GMP baseline to 52 wk

Wilcoxon test §§ L-AA baseline to 26 wk., ** L-AA baseline to 52 wk

ABREVIATIONS: *CGMP-AA* low phenylalanine glycomacropeptide protein substitute supplemented with L-amino acids, *Phe-free L-AA* Phenylalanine free L-amino acid supplement

**Table 4:** Changes in the median (range) blood Phe:Tyr ratio within and between the CGMP-AA2 and Phe-free L-AA group at baseline, 26 and 52 weeks

Group	Number of subjects	Baseline (range)	Wk 26 (range)	Wk 52 (range)	P value
CGMP-AA2	29	5.7^§*^ (1.5–11)	6.4^§^ (1.3–11.7)	6.4^*^ (1.7–11.5)	^§^p = 0.010 ^*^p < 0.010
Phe-free L-AA	19	7.2^§§**^ (3.1–15)	6.8^§§^ (3–11.7)	7.4^**^ (4–15.1)	^§§^*p* = 0.555 ^**^p = 0.246

Wilcoxon test § GMP baseline to 26 wk., * GMP baseline to 52 wk

Wilcoxon test §§ L-AA baseline to 26 wk., ** L-AA baseline to 52 wk

ABREVIATIONS: *CGMP-AA* low phenylalanine glycomacropeptide protein substitute supplemented with L-amino acids, *Phe-free L-AA* Phenylalanine free L-amino acid supplement

For each group the median Phe and Tyr values were calculated for each subject over a time period from baseline to week 26, and from week 27 to 52. The median of the collective medians has been reported.

#### Blood Phe levels

Phe levels are consistently higher in the L-AA group compared with the CGMP-AA2 group. Both groups observed a rise in Phe levels over time, but this rise was more pronounced in the CGMP-AA2 group with a statistical difference between baseline and 52 weeks (*p* < 0.001).

#### Blood Tyr levels

Whilst Tyr levels were consistently higher in the CGMP-AA2 groups, there were no significant differences between the CGMP-AA2 and L-AA groups and no differences in the change in Tyr over time.

#### Phe: Tyr ratio

For the CGMP-AA2 group there was a small but consistent rise in the Phe:Tyr ratio with the difference between baseline and 26 weeks (*p* = 0.010) and baseline and 52 weeks (p < 0.001) both statistically significant. Changes in the Phe:Tyr ratio in the L-AA group were not statistically significant.

### Sub- group analysis for children < 12 years of age (Table 5) [Additional file 1]

We compared blood Phe concentrations over the same time period in children aged < 12 years in both groups. There were 25 children in the CGMP-AA2 group (median age 8.9y), and 9 children in the Phe-free L-AA group (median age 9.2y). There were no significant differences between the groups for median blood Phe at baseline, 26 or 52 weeks. However, a significant difference was observed for median blood Phe in the CGMP-AA2 group between baseline and week 26 (*p* = 0.022) and baseline and week 52 (p = 0.010). No differences were observed at any time point in the Phe-free L-AA group.

**Table 5:** Changes in the median (range) blood Phe concentrations (μmol/L) within and between the CGMP-AA2 and Phe-free L-AA group at baseline, 26 and 52 weeks for children < 12 years

Group	Number of subjects	Baseline μmol/L (range)	Wk 26 μmol/L (range)	Wk 52 μmol/L (range)	P value
CGMP-AA2	25	270 ^§*^ (170–360)	300^§^ (125–415)	290^*^ (200–450)	^§^*p* = 0.04 ^*^p = 0.04
Phe-free L-AA	9	288 ^§§**^ (140–470)	287^§§^ (180–348)	291^*^ (190–344)	^§^*P* = 0.90 ^**^p = 0.41

Wilcoxon test § GMP baseline to 26 wk., * GMP baseline to 52 wk

Wilcoxon test §§ L-AA baseline to 26 wk., ** L-AA baseline to 52 wk

ABABBREVIATIONS: *CGMP-AA* low phenylalanine glycomacropeptide protein substitute supplemented with L-amino acids, *Phe-free L-AA* Phenylalanine free L-amino acid supplement

### Anthropometry (Fig. 3)

#### Comparison of weight, height and BMI z scores between the CGMP-AA 2 and Phe-free L-AA groups and within groups at: Baseline, week 26 and 52

There was no statistical significance for median weight, height or BMI between the CGMP-AA2 and L-AA group at each of the measured time points. However, in the CGMP-AA2 group a significant increase was observed for median (range), weight z score from baseline (0.55, − 1.93-2.34) to week 52 (0.77, − 1.75-2.60), (p = < 0.0001) and BMI z score from baseline (0.58, range − 0.88-2.83) to week 52 (0.92 range, − 0.67-2.98), (p = < 0.0001). A significant increase was also observed from week 26 (0.53 range, − 1.8-2.47) to 52 (0.77, range − 1.75-2.60) for weight z score (p = < 0.0001), and BMI z score between week 26 (0.61, range − 1.9-2.81) and week 52 (0.92 range, − 0.67-2.98) (p = < 0.0001).

**Fig. 3:**
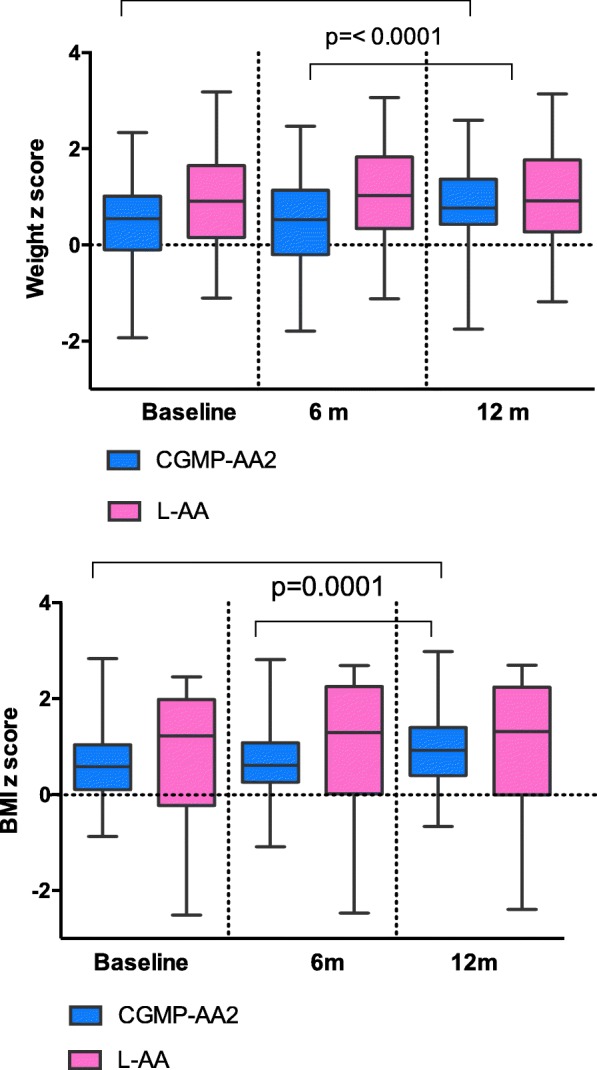
Weight and BMI z scores for CGMP-AA2 and L-AA at baseline, 26 and 52 weeks

In the Phe-free L-AA group there was no significant differences for median weight or BMI z scores: baseline to week 52 (weight z score: [*p* = 0.11]; BMI z score, [*p* = 0.14]) or from 26 to 52 weeks: (weight z score, [*p* = 0.80]; BMI z score, [*p* = 0.32]). However, at baseline the control group had a median weight, height and BMI that were nearly double that of the CGMP-AA2 group.

### Nutritional biochemistry (Table 6)

#### Comparison of nutritional biochemistry between the CGMP-AA2 and Phe-free L-AA groups and within groups at baseline and 26 weeks

The median values for all nutritional parameters measured at baseline and 26 weeks were all within the reference ranges. One exception, in both groups was vitamin B12, which was higher than the reference range at 26 weeks. No differences were found between any of the parameters at baseline when all the children were taking Phe-free L-AA.

**Table 6:** Median nutritional biochemistry comparing CGMP-AA2 with Phe-free L-AA at baseline and week 26

Parameters	Reference range		Base-line (range)		Significance Base-line	26wk (range)		Significance at 26wk
			Phe-free L-AA	CGMP-AA2		Phe-free L-AA	CGMP-AA2	
Zinc μmol/L	11–24		14.3 (12.9–17.4)	14.4 (11.3–18.2)	p = 0.3	14.55 (12.3–17.5)	14.0 (10.5–16.4)	*p* = 0.24
C reactive protein mg/L	< 10		1.0 (1–4)	1.0 (1–11)	*p* = 0.27	1.0 (1–3)	1.0 (1–6)	*p* = 0.69
Whole blood Selenium	ns		0.94 (0.76–1.21)	0.99^§^ (0.48–1.38)	*p* = 0.57	0.98^*^ (0.75–1.23)	1.22^*§^ (0.86–1.56)	^**§**^ **p = < 0.0001** ^*^ **p = 0.0002**
Selenium μmol/L	**4-7y**	0.7–1.7	0.86 (0.53–1.1)	0.91^§^ (0.38–1.4)	p = 0.57	0.85^*^ (0.58–1.14)	1.12^*§^ (0.58–1.35)	^**§**^ **p = 0.0005** ^*^ **p = 0.0007**
	**7-18y**	0.62–1.3						
Calcium mmol/L	2.2–2.7		2.46 (2.33–2.56)	2.49 (2.32–2.63)	*p* = 0.56	2.44 (2.31–2.59)	2.45 (2.25–2.68)	*p* = 0.77
Magnesium mmol/L	0.7–1		0.84 (0.75–0.92)	0.85 (0.72–1.0)	p = 0.68	0.82 (0.72–0.92)	0.84 (0.76–0.92)	p = 0.14
Phosphate mmol/L	0.9–1.8		1.30 (0.94–1.53)	1.38 (1.0–1.59)	*p* = 0.08	1.33 (1.0–1.58)	1.39 (1.0–1.71)	*p* = 0.20
25 hydroxy vitamin D nmol/L	> 50		93.9 (38.8–182.3)	95.3 (60–162)	p = 0.27	84.2 (47.6–139)	87.6 (47.9–158)	*p* = 0.49
Haemoglobin g/L	120–140		137 (119–164)	136 (101–156)	p = 0.55	141 (120–164)	135 (109–155)	p = 0.23
MCV fl	78–93		82 (76–90)	82 (75–91)	*p* = 0.48	84 (77–93)	82.5 (76–88)	*p* = 0.50
Ferritin μg/L	11–60		32.8 (20.9–63.1)	27.4^§§^ (17.9–116.5)	*p* = 0.16	30.80 (17.6–75.6)	25.2^§§^ (15.9–91.6)	^§§^ **p = 0.0006**
Vitamin B12ng/L	245–798		798 (390–1500)	1088 (333–1500)	p = 0.1	745 (308–1500)	922 (437–1500)	*p* = 0.23

ns not specified

Wilcoxon test (paired and unpaired t test)

^§^Whole blood and plasma selenium CGMP-AA2 baseline compared with CGMP-AA2 at 26 weeks

*Whole blood and plasma selenium CGMP-AA2 26 weeks compared with L-AA at 26 weeks

^§§^ Ferritin CGMP-AA2 baseline compared with CGMP-AA2 at 26 weeks

Whole blood and plasma selenium were significantly higher (whole blood selenium [*p* = 0.0002]; plasma selenium [*p* = 0.0007]) at 26 weeks in the CGMP-AA2 group compared L-AA.

Within the CGMP-AA2 group between baseline and week 26, whole blood and plasma selenium significantly increased (whole blood selenium [p = < 0.0001]; plasma selenium [*p* = 0.0005]) and ferritin decreased (*p* = 0.0006). Median values all remained within the reference ranges.

## Discussion

This is the first longitudinal, comparative prospective study over 12 months reporting the use of CGMP-AA2 compared to conventional protein substitute L-AA in children with PKU. After 12 months of using the modified CGMP-AA2 there were no differences for Phe, Tyr, Phe:Tyr ratio and anthropometry compared to the control group using L-AA. However, in the same period within the CGMP-AA2 group, blood Phe concentrations significantly increased although this was only by 30 μmol/L. When comparing children < 12 years of age, within the CGMP-AA2 group, the same small but consistent significant increase for blood Phe concentrations were evident. Weight and BMI z scores significantly increased within the CGMP-AA2 group. Plasma and whole blood selenium improved although ferritin decreased but all nutritional measurements remained within the reference ranges. Identifying the reason for these physical and biochemical changes over 12 months in the CGMP-AA2 group is important to assess the suitability of using CGMP-AA2 as a protein substitute in children with PKU.

We have previously reported a small but significant increase in blood Phe concentrations in the first group of children recruited to the pilot study using CGMP-AA1 [11]. CGMP-AA1 was based on an AA profile that met the minimum safe levels of amino acid intake (WHO/FAO/UNU 2007) [13] for Tyr, tryptophan, leucine, and histidine. CGMP-AA1 contained 30 mg of Phe for every 20 g protein equivalent. A modified CGMP-AA formula (CGMP-AA2) was produced by making small adjustments to the AA composition of CGMP-AA1, increasing some of the LNAA (Tyr, tryptophan, leucine and histidine) in amounts similar to conventional L-AA supplements. Although in this study, we examined a different cohort of children, after using CGMP-AA2 for 12 months, blood Phe concentrations, were lower than those in the pilot study using CGMP-AA1. Median Phe concentrations at the end of the pilot study 317 μmol/L compared to CGMP-AA2 after 12 months Phe 300 μmol/L. We were also able to increase the amount of protein equivalent supplied from CGMP-AA2 to 75% of total protein equivalent, without any reduction in dietary Phe intake. However, at the end of this study period, only 14 of 29 children (48%) were able to fully transition to CGMP-AA2 as their only protein substitute, which suggests that the residual Phe concentration present in CGMP-AA2 still increases blood Phe concentrations in children.

Identifying the optimal amino acid profile for protein substitutes is challenging, although, the ratio and amount of LNAA appears important. It is suggested that LNAA supplementation competes with Phe uptake at the gut and blood brain barrier (BBB): LNAA cross the intestinal mucosa using a carrier protein similar to that at the BBB [14–16]. In vitro studies investigating intestinal epithelial transport of amino acids, indicate that lysine, histidine, leucine and Tyr significantly reduce Phe uptake [6]. High concentrations of LNAA compete with the transport of Phe at the gut cellular membrane and may decrease blood Phe concentrations. Additionally LNAA supplementation has been shown to reduce blood and brain Phe concentrations [17, 18] and restore some of the disturbed Phe transport across the BBB by altering monoaminergic neurotransmitter concentrations. In mice studies, mice chow with added LNAA, improved brain tryptophan, serotonin and norepinephrine concentrations [4]. There are other functional effects of protein substitutes, which indirectly affect blood Phe concentrations. These include the rate of delivery of L-AA into the systemic circulation and muscle protein anabolism. Amino acids play an important, but as yet not fully understood role in nutritional signalling and regulation of multiple cellular processes [19]. Leucine, is a potential insulin secretagogue when administered with carbohydrate and protein, acting as a pharmaconutrient AA improving muscle protein synthesis, by stimulating mRNA changes via insulin independent and dependent pathways [7, 20]. Van Loon et al. maximised endogenous insulin secretion by the combined ingestion of carbohydrate and wheat protein hydrolysate, with added leucine and Phe [21]. In vitro studies, using incubated β cells of the pancreas, have shown that arginine, leucine and phenylalanine have a strong insulinotropic effect [22]. Further studies are needed to maximize our understanding of the physiological absorption process of AA protein substitutes, with the goal, to achieve a normal absorption pattern comparable to natural intact protein.

In contrast, many L-AA are bitter tasting and unpleasant to take, particularly leucine, tryptophan and histidine [23, 24]. By adding more of these AA to CGMP-AA formulations, it potentially decreases their acceptability and palatability. It has been suggested by Van Calcar [25] that the AA profile in CGMP-AA should provide 130 to 150% of the 2002 Institute of Medicine American dietary reference intake for histidine, leucine, methionine, tryptophan and Tyr to compensate for faster absorption and degradation of AA [26].

In our study, 52% of children in the CGMP-AA2 group were prescribed a combination of CGMP-AA2 and separate Phe-free L-AA supplements, as they could not fully transition onto CGMP-AA2 in order to maintain blood Phe concentrations within the target range of 120 to 360 μmol/L. The median amount of protein substitute provided by CGMP-AA2 that could be tolerated without affecting blood Phe control was 75% of the total amount. This contrasts with other researchers findings that have reported that the Phe content of CGMP-AA has little effect on blood Phe concentration. In an uncontrolled, short term study reported in 10 children (aged 4 to 16 years) with PKU, when 50% of their total protein requirements were supplied by ‘GMP cheese’ for 9 weeks, blood Phe concentrations decreased by a median of 114 μmol/L although this was not statistically significant [27]. In a short-term randomized cross over trial in 30 patients aged 15 years or over, comparing CGMP-AA with L-amino acid supplements only, CGMP-AA was associated with a non-significant increase in Phe of 62 ± 40 μmol/L, although 10 of the 30 patients were prescribed sapropterin (likely to have improved Phe tolerance), 6 patients appeared less adherent with CGMP-AA and overall subjects were only studied over a short time and had a higher baseline blood Phe levels in comparison to our study group [28]. Furthermore, increases of between 60 to 102 μmol/L may be unacceptable in children particularly as evidence is accumulating to suggest that optimal blood Phe may be below 240 μmol/L [29].

Median nutritional blood parameters in both groups at baseline and 26 weeks were all within reference ranges, with the exception on vitamin B12 being higher than the reference range at 26 weeks in both groups. There were no biochemical signs of vitamin or mineral deficiencies in line with what was retrospectively reported in adult PKU patients [30]. There was a significant increase in whole blood and plasma selenium between the groups at 26 weeks, and within the CGMP-AA2 group from baseline to 26 weeks. The selenium content of both products was similar, a median intake of 60 g protein equivalent from Phe-free L-AA provided 87 mg of selenium compared with CGMP-AA2 providing 90 mg. It is only conjecture, but absorption of selenium in the CGMP-AA2 group may be enhanced based on its bioactive properties. It is also possible that CGMP may modulate microbiota resulting in a different absorption or bioavailability. Furthermore, whey protein is rich in the sulfhydryl amino acid cysteine, which is a precursor of glutathione, and may in part explain the higher selenium concentrations in the CGMP-AA2 group. Muniz-Naveiro [31] reported the largest percentage of selenium in cow’s milk was found in the whey phase, although unmodified CGMP is not high in cysteine or selenium. Peptides containing isoleucine, proline, lysine, glutamine, aspartic and glutamic acid have been shown to have antioxidant properties; hence peptide structure and amino acid sequencing influence biological function. The bioactive antioxidant properties of CGMP and absorption in the gut may have a selenium sparing effect compared to protein substitutes without a peptide base [32, 33].

Weight and BMI increased significantly in the CGMP-AA2 group with an increase in weight and BMI first becoming evident from 26 weeks, this may be related to some children using a protein free milk replacement to make up their powdered CGMP-AA2 protein substitute. A mean intake of 400 ml/day of protein-free milk replacement would increase energy intake by 270 kcal/day. Only 9 children were routinely adding additional milk substitute to their CGMP-AA2 but there were no significant differences evident between children using protein free milk or water to prepare the CGMP-AA 2, but this may not be apparent due to the small numbers in the group. We changed this practice when it was observed that children were gaining extra weight. Another consideration influencing growth is age; the Phe-free L-AA group were an older patient cohort and therefore, over the 12-month period it is difficult to quantify how many were actively reaching puberty and the effect this has on weight, height and BMI. A further consideration is that CGMP based on a protein source may be more efficiently utilized increasing muscle mass compared to L-AA. Longer-term observations of both study groups will hopefully answer this question with data describing both fat free and fat mass index.

There are several limitations to this study that need to be considered. We were unable to conduct a randomised controlled blind trial. CGMP-AA2 and conventional L-AA supplements are very different in taste, texture and appearance rendering any blind or randomised trial very challenging in children who may not easily accept changes to their protein substitutes [34]. In this study, 40% of children preferred to stay on their usual Phe-free L-amino acid supplement (control group) and so they were a self-selected, not age-matched group and children in the control group were older than the study group. Also in teenage children in both study groups, ensuring dietary adherence was difficult although patients were monitored closely with monthly home visits to check stock levels of protein substitute and dietary intake. A further limitation was the use of low protein milk substitute to make up the protein substitute, in addition to increasing energy intake it possibly altered CGMP-AA2 absorption, affecting amino acid kinetics.

## Conclusions

It is clear that the biological functions of CGMP may potentially offer many health benefits in PKU [35–40] and it is likely to play a significant future role in provision of low Phe protein substitute. However, it is important that the manufacturers of CGMP seek to reduce its Phe content and the formulation of CGMP-AA2 requires further research and development in order to ensure optimal amino acid profiling.

## Additional file

Additional file 1: Supplementary data using mixed linear models to express measured Phe, Tyr and Phe: Tyr ratio in the CGMP-AA2 and L-AA groups. **Figure S1A, B and C** showing mixed linear models for measured Phe, Tyr, Phe:Tyr ratio for CGMP-AA2 and L-AA groups and mean 95% confidence intervals. **Figure S2.** showing Mixed linear model for measured Phe levels in children < 12 years of age in the CGMP-AA2 and L-AA groups and mean 95% confidence intervals. (DOCX 823 kb)

## Abbreviations

CGMP-AA: Casein glycomacropeptide – amino acids
GMP: Glycomacropeptide
L-AA: L-amino acids
PKU: Phenylketonuria
