# Supplementary material for: Glycomacropeptide: long-term use and impact on blood phenylalanine, growth and nutritional status in children with PKU
Source: Orphanet J Rare Dis. 2019 Feb 15;14:44. doi: 10.1186/s13023-019-1011-y (PMC6377744; doi:10.1186/s13023-019-1011-y)
Supplement: Supplementary file 1 — Supplementary data using mixed linear models to express measured Phe, Tyr and Phe: Tyr ratio in the CGMP-AA2 and L-AA groups. Figure S1A, B and C showing mixed linear models for measured Phe, Tyr, Phe:Tyr ratio for CGMP-AA2 and L-AA groups and mean 95% confidence intervals. Figure S2. showing Mixed linear model for measured Phe levels in children < 12 years of age in the CGMP-AA2 and L-AA groups and mean 95% confidence intervals. (DOCX 823 kb) [file 13023_2019_1011_MOESM1_ESM.docx]

**Figure S1A** Mixed linear models for measured Phe for CGMP-AA2 and L-AA groups and mean 95% confidence intervals.

*

*

CGMP-AA2 * p <0.001

L-AA

**Figure S1B** Mixed linear models for measured Tyr for CGMP-AA2 and L-AA groups and mean 95% confidence intervals.

CGMP-AA2

L-AA

**Figure S1C** Mixed linear models for measured Phe/Tyr ratio for CGMP-AA2 and L-AA groups and mean 95% confidence intervals.

§ **

**

§

CGMP-AA2 § p= 0.01, **p< 0.01

L-AA

**Figure S2** Mixed linear models for measured Phe levels in children < 12 years of age in the CGMP-AA2 and L-AA groups and mean 95% confidence intervals.

CGMP-AA2 § p=0.022, ** p= 0.010

**

§

§ **

L-AA
